# Comparative Effects of IL-17 and IL-23 Inhibitors on Lipid Profiles and Lipid-Derived Atherogenic Indices in Patients with Psoriasis

**DOI:** 10.3390/ph19101624

**Published:** 2026-10-13

**Authors:** Candan Celik, Mehmet Semih Çelik, Nagehan Cepik

**Affiliations:** 1 Department of Dermatology, Malatya Training and Research Hospital, 44330 Malatya, Turkey; 2 Department of Dermatology, Diyarbakır Gazi Yaşargil Training and Research Hospital, University of Health Sciences, 21070 Diyarbakir, Turkey

**Keywords:** psoriasis, IL-23 inhibitors, IL-17 inhibitors, atherogenic index of plasma, lipid-derived atherogenic indices, cardiovascular risk

## Abstract

**Background:** Psoriasis is associated with increased cardiovascular risk and altered lipid metabolism. The comparative effects of IL-17 and IL-23 inhibitors on lipid-derived atherogenic indices remain unclear. **Objective:** To compare the effects of IL-17 and IL-23 inhibitors on lipid parameters and lipid-derived atherogenic indices in patients with psoriasis. **Methods:** In this retrospective cohort study, 152 patients were assessed for eligibility, and 108 were included: 51 receiving IL-23 inhibitors and 57 receiving IL-17 inhibitors. Lipid parameters, lipid-derived indices, and PASI were evaluated at baseline and after 12 months. **Results:** Both groups achieved marked PASI improvement. IL-23 inhibitors were associated with significant reductions in total cholesterol, non-HDL-C, triglycerides, VLDL-C, remnant cholesterol, AIP, TG/HDL-C ratio, CRI-I, CRI-II, and AC, whereas only the TG/HDL-C ratio decreased significantly in the IL-17 group. Between-group comparisons showed greater reductions with IL-23 inhibitors in these parameters and indices (all *p* < 0.05), while changes in PASI, LDL-C, and HDL-C were comparable. **Conclusion:** IL-23 inhibitors were associated with greater improvements in several lipid parameters and lipid-derived atherogenic indices than IL-17 inhibitors despite comparable clinical efficacy. These findings should not be interpreted as evidence of differential cardiovascular protection.

## 1. Introduction

Psoriasis is a chronic immune-mediated inflammatory skin disease affecting approximately 2–3% of the global population and substantially impairing patients’ quality of life. Plaque psoriasis is the most common clinical subtype, accounting for nearly 80–90% of all cases [1,2]. Increasing evidence has established that psoriasis is a systemic inflammatory disorder rather than a disease confined to the skin. Persistent immune dysregulation and chronic inflammation contribute to the development of multiple comorbidities, including psoriatic arthritis, obesity, metabolic syndrome, type 2 diabetes mellitus, nonalcoholic fatty liver disease, and cardiovascular disease, resulting in a substantially increased cardiometabolic burden compared with the general population [3,4,5]. Psoriasis pathogenesis involves a complex interplay among genetic susceptibility, immune dysregulation, and environmental factors, ultimately leading to sustained activation of innate and adaptive immune responses. This inflammatory cascade promotes cytokine-mediated keratinocyte activation and hyperproliferation, which in turn amplify and maintain the chronic inflammatory process. Among the inflammatory pathways involved in psoriasis, the IL-23/IL-17 axis plays a pivotal role in disease pathogenesis. IL-23, predominantly produced by activated antigen-presenting cells, promotes the expansion, maintenance, and pathogenicity of Th17 cells, leading to increased production of IL-17A, IL-17F, IL-22, and other pro-inflammatory mediators that drive keratinocyte activation and epidermal hyperproliferation.

Advances in the understanding of the IL-23/IL-17 pathway, together with insights gained from the clinical efficacy of targeted biologic therapies, have substantially improved current knowledge of psoriasis pathogenesis and supported the development of highly effective biologic agents targeting IL-23 or IL-17. Although both therapeutic classes provide substantial clinical improvement, their effects on systemic inflammation and cardiometabolic biomarkers may not be identical [1,6,7,8]. Patients with psoriasis have an increased risk of cardiovascular disease compared with the general population, and this association becomes stronger with increasing disease severity and cumulative inflammatory burden. Chronic systemic inflammation, endothelial dysfunction, oxidative stress, and disturbances in lipid metabolism contribute to accelerated atherosclerosis in these patients. Consequently, cardiovascular risk assessment has become an important component of psoriasis management. Beyond conventional lipid parameters, lipid-derived cardiovascular risk markers have gained increasing attention as complementary tools for cardiometabolic risk assessment. Among these, the atherogenic index of plasma (AIP) has been the most extensively investigated and has shown significant associations with coronary artery disease and a broad spectrum of metabolic disorders, as reported in recent narrative reviews, systematic reviews, and meta-analyses [9,10,11]. Although biologic therapies targeting the IL-23 and IL-17 pathways have revolutionized the treatment of moderate-to-severe psoriasis, comparative evidence regarding their effects on lipid-derived cardiovascular risk markers remains limited [12]. Therefore, the present study aimed to compare the effects of IL-23 and IL-17 inhibitors on conventional lipid parameters and a comprehensive panel of lipid-derived cardiovascular risk markers, including the atherogenic index of plasma, triglyceride-to-high-density lipoprotein cholesterol ratio, Castelli risk indices, atherogenic coefficient, and remnant cholesterol, in patients with moderate-to-severe psoriasis.

## 2. Results

### 2.1. Baseline Characteristics

A total of 108 patients were included in the study, comprising 57 patients treated with IL-17 inhibitors and 51 patients treated with IL-23 inhibitors. Baseline demographic, clinical, and lipid-related characteristics are summarized in Table 1. The mean age was 45.9 ± 9.81 years in the IL-17 inhibitor group and 44.1 ± 10.9 years in the IL-23 inhibitor group, with no significant difference between the groups (*p* = 0.367). Similarly, the proportion of male patients was comparable between the IL-23 and IL-17 inhibitor groups (64.7% [33/51] vs. 63.2% [36/57], *p* = 0.867). Similarly, baseline PASI scores were comparable (18.8 ± 4.67 vs. 19.2 ± 4.57, *p* = 0.701). At baseline, patients receiving IL-17 inhibitors had significantly higher total cholesterol (200 ± 27.6 vs. 189 ± 26.2 mg/dL, *p* = 0.039) and LDL cholesterol (123 ± 23.3 vs. 111 ± 25.9 mg/dL, *p* = 0.013) than those receiving IL-23 inhibitors. In contrast, HDL cholesterol (44.2 ± 7.72 vs. 44.0 ± 11.5 mg/dL, *p* = 0.929), non-HDL cholesterol (156 ± 29.1 vs. 145 ± 28.7 mg/dL, *p* = 0.059), triglycerides (164 ± 56.0 vs. 154 ± 60.7 mg/dL, *p* = 0.138), and VLDL cholesterol (32.7 ± 11.2 vs. 30.7 ± 12.1 mg/dL, *p* = 0.111) did not differ significantly between the two groups. Likewise, baseline lipid-derived atherogenic indices were comparable between treatment groups, including AIP (0.185 ± 0.225 vs. 0.170 ± 0.241, *p* = 0.721), TG/HDL ratio (3.98 ± 2.18 vs. 3.95 ± 2.43, *p* = 0.714), Castelli Risk Index-I(4.68 ± 1.12 vs. 4.64 ± 1.58, *p* = 0.293), Castelli Risk Index-II (2.89 ± 0.77 vs. 2.71 ± 0.93, *p* = 0.275), atherogenic coefficient (3.68 ± 1.12 vs. 3.64 ± 1.58, *p* = 0.293), and remnant cholesterol (32.5 ± 11.2 vs. 33.8 ± 18.5 mg/dL, *p* = 0.588) (Table 1). Given the significant baseline differences in total cholesterol and LDL-C between the treatment groups, additional ANCOVA analyses were performed using the corresponding baseline value as a covariate. After adjustment for baseline levels, no statistically significant between-group differences were observed at month 12 for total cholesterol (*p* = 0.076) or LDL-C (*p* = 0.543).

### 2.2. IL-23 Inhibitor Group

After 12 months of treatment, significant reductions were observed in total cholesterol (189.06 ± 26.18 vs. 182.12 ± 26.89 mg/dL, *p* = 0.013), non-HDL cholesterol (145.07 ± 28.72 vs. 134.62 ± 31.02 mg/dL, *p* < 0.001), triglycerides (154.06 ± 60.75 vs. 136.24 ± 52.04 mg/dL, *p* < 0.001), VLDL cholesterol (30.70 ± 12.07 vs. 27.36 ± 10.45 mg/dL, *p* = 0.005), remnant cholesterol (33.79 ± 18.51 vs. 24.91 ± 15.70 mg/dL, *p* = 0.001), AIP (0.170 ± 0.241 vs. 0.090 ± 0.243, *p* < 0.001), TG/HDL ratio (3.95 ± 2.43 vs. 3.24 ± 1.67, *p* < 0.001), Castelli Risk Index-I (4.64 ± 1.58 vs. 4.19 ± 1.08, *p* = 0.007), Castelli Risk Index-II (2.71 ± 0.93 vs. 2.56 ± 0.75, *p* = 0.043), and atherogenic coefficient (3.64 ± 1.58 vs. 3.19 ± 1.08, *p* = 0.007). No significant changes were detected in LDL cholesterol (111.28 ± 25.87 vs. 110.81 ± 21.48 mg/dL, *p* = 0.381) or HDL cholesterol (44.00 ± 11.46 vs. 46.39 ± 13.98 mg/dL, *p* = 0.168). In addition, disease activity markedly improved following treatment, with the mean PASI score decreasing from 19.16 ± 4.57 to 0.91 ± 1.09 (*p* < 0.001) (Table 2).

### 2.3. IL-17 Inhibitor Group

After 12 months of treatment, no significant changes were observed in total cholesterol (199.90 ± 27.62 vs. 198.67 ± 34.15 mg/dL, *p* = 0.123), LDL cholesterol (123.23 ± 23.33 vs. 121.94 ± 28.85 mg/dL, *p* = 0.144), HDL cholesterol (44.16 ± 7.72 vs. 45.34 ± 7.82 mg/dL, *p* = 0.188), non-HDL cholesterol (155.73 ± 29.13 vs. 153.33 ± 35.55 mg/dL, *p* = 0.080), triglycerides (163.54 ± 55.99 vs. 164.09 ± 61.35 mg/dL, *p* = 0.100), VLDL cholesterol (32.74 ± 11.19 vs. 32.95 ± 12.36 mg/dL, *p* = 0.067), AIP (0.185 ± 0.225 vs. 0.179 ± 0.193, *p* = 0.066), Castelli Risk Index-I (4.68 ± 1.12 vs. 4.51 ± 1.11, *p* = 0.138), Castelli Risk Index-II (2.89 ± 0.77 vs. 2.78 ± 0.86, *p* = 0.054), atherogenic coefficient (3.68 ± 1.12 vs. 3.51 ± 1.11, *p* = 0.139), or remnant cholesterol (32.50 ± 11.24 vs. 31.39 ± 12.09 mg/dL, *p* = 0.481). However, a significant reduction was observed in the TG/HDL ratio (3.98 ± 2.18 vs. 3.80 ± 1.76, *p* = 0.034). Disease activity also improved markedly following treatment, with the mean PASI score decreasing from 18.82 ± 4.67 to 0.86 ± 0.97 (*p* < 0.001) (Table 3).

### 2.4. Comparison of Changes Between Treatment Groups

Changes (Δ) in lipid parameters and lipid-derived atherogenic indices were compared between the IL-23 and IL-17 groups. Significant between-group differences were observed for ΔTC (*p* = 0.001), ΔNon-HDL (*p* < 0.001), ΔTG (*p* < 0.001), ΔVLDL (*p* < 0.001), ΔAIP (*p* < 0.001), ΔTG/HDL ratio (*p* < 0.001), ΔCRI-I (*p* = 0.002), ΔCRI-II (*p* = 0.015), ΔAC (*p* = 0.002), and ΔRemnant cholesterol (*p* < 0.001). In contrast, changes in LDL cholesterol (*p* = 0.117), HDL cholesterol (*p* = 0.233), and PASI score (*p* = 0.739) were comparable between the two treatment groups (Table 4).

## 3. Discussion

The principal finding of the present study is that IL-23 and IL-17 inhibitors were associated with distinct changes in lipid metabolism and lipid-derived cardiovascular risk markers in patients with moderate-to-severe psoriasis. Although both therapeutic classes achieved comparable clinical improvement, as reflected by similar reductions in PASI scores, IL-23 inhibitors were associated with greater improvements in several conventional lipid parameters and lipid-derived cardiovascular risk markers than IL-17 inhibitors. These findings suggest that biologic therapies targeting different inflammatory pathways may be associated with differential changes in lipid-derived cardiometabolic risk indices, despite comparable efficacy in controlling cutaneous inflammation. Importantly, baseline total cholesterol and LDL-C levels differed significantly between the treatment groups. However, additional ANCOVA analyses adjusting for the corresponding baseline values showed no significant between-group differences in total cholesterol or LDL-C at month 12. These findings indicate that the observed unadjusted differences in lipid changes should be interpreted cautiously, particularly in the presence of baseline imbalance.

The differential changes observed between IL-23 and IL-17 inhibitors may be biologically plausible considering the distinct roles of these cytokines within the IL-23/IL-17 inflammatory axis. IL-23 functions upstream by promoting the differentiation, expansion, and maintenance of pathogenic Th17 cells, whereas IL-17 acts as a downstream effector cytokine involved in keratinocyte activation and amplification of local inflammation. These biological differences may potentially contribute to the distinct changes observed in lipid metabolism and lipid-derived cardiovascular risk markers. However, the present study was not designed to investigate the mechanisms underlying these differences, and therefore this interpretation should be considered hypothesis-generating [6,7,8,9].

Previous studies evaluating the cardiometabolic effects of biologic therapy in psoriasis have yielded heterogeneous results. Although several investigations have reported favorable effects on inflammatory activity and cardiovascular risk-related parameters, the magnitude and direction of changes in lipid metabolism have varied according to biologic class, treatment duration, and the metabolic parameters assessed. Available evidence suggests that both IL-17 and IL-23 inhibitors may influence metabolic homeostasis beyond their effects on skin clearance; however, their effects on conventional lipid parameters and lipid-derived cardiometabolic indices remain inconsistent across studies. Large cohort studies have also reported lower cardiovascular event rates among patients receiving biologic therapy, supporting a potential relationship between suppression of systemic inflammation and cardiovascular risk, although direct cardiovascular protection attributable to specific biologic classes has not been established. In contrast, some retrospective studies have demonstrated marked improvement in disease activity without significant changes in conventional lipid parameters, indicating that standard lipid measurements alone may not fully capture treatment-related metabolic changes. Furthermore, metabolomic studies have identified broader alterations in lipid metabolism during biologic therapy, suggesting a complex interaction between immune modulation and metabolic regulation [13,14,15,16,17].

Recent studies have increasingly focused on lipid-derived atherogenic and cardiometabolic risk indices rather than conventional lipid parameters alone in patients with psoriasis. A large population-based NHANES analysis demonstrated that higher AIP values were independently associated with the presence of psoriasis, supporting the potential role of AIP as a surrogate marker of atherogenic dyslipidemia in this population. Similarly, Aksoy et al. reported significantly higher AIP, Castelli Risk Index I and II, and atherogenic coefficient values in patients with psoriasis compared with healthy controls, indicating a more atherogenic lipid profile. More recently, Yıldız et al. showed that six months of IL-23 inhibitor therapy significantly reduced both AIP and the triglyceride-glucose (TyG) index, suggesting favorable changes in lipid-derived cardiometabolic risk markers during treatment. Collectively, these findings indicate that lipid-derived indices may provide additional information regarding disease-associated cardiometabolic burden and treatment-related metabolic changes. In line with these observations, our study extends the current evidence by simultaneously evaluating a broader panel of lipid-derived atherogenic indices, including the TG/HDL-C ratio, Castelli risk indices, atherogenic coefficient, non-HDL-C, remnant cholesterol, and AIP, while directly comparing IL-17 and IL-23 inhibitors within the same patient population [18,19,20].

### Limitations

This study has several limitations. First, its retrospective design may have introduced selection bias and limits the ability to establish causal relationships. Second, the follow-up period was limited to 12 months; therefore, the longer-term effects of IL-17 and IL-23 inhibitors on lipid-derived atherogenic and cardiometabolic risk markers could not be evaluated. Third, although a comprehensive panel of conventional lipid parameters and lipid-derived indices was assessed, the study did not include hard cardiovascular outcomes, such as myocardial infarction, stroke, or cardiovascular mortality. In addition, inflammatory biomarkers were not systematically assessed; therefore, the observed lipid changes cannot be directly attributed to differential modulation of the IL-23/IL-17 inflammatory axis, and the proposed mechanistic explanations should be regarded as hypothesis-generating.

Although patients with major conditions or treatments known to substantially affect lipid metabolism were excluded to reduce potential confounding, residual confounding inherent to the retrospective design cannot be completely excluded. Furthermore, the exclusion of patients with major comorbidities or treatments known to substantially affect lipid metabolism resulted in a relatively selected study population. Therefore, the generalizability of our findings to the broader psoriasis population, in whom cardiometabolic comorbidities are common, may be limited. Moreover, detailed information on dietary habits and changes in diet during the follow-up period was not systematically available because of the retrospective study design. Therefore, the potential influence of dietary factors on lipid parameters and lipid-derived atherogenic indices could not be fully accounted for. Finally, multiple statistical comparisons were performed without formal adjustment for multiple testing; therefore, particularly the findings with modest statistical significance should be interpreted cautiously and confirmed in larger prospective studies.

## 4. Materials and Methods

### 4.1. Study Design and Patients

This retrospective observational study included adult patients with moderate-to-severe plaque psoriasis who were followed at the Dermatology Outpatient Clinic and received treatment with either IL-23 or IL-17 inhibitors. A total of 152 patients were initially assessed for eligibility. After application of the predefined inclusion and exclusion criteria, 108 patients were included in the final analysis, including 51 patients treated with IL-23 inhibitors and 57 patients treated with IL-17 inhibitors. The IL-17 inhibitor group comprised 33 patients treated with secukinumab and 24 patients treated with bimekizumab, whereas the IL-23 inhibitor group comprised 29 patients treated with risankizumab and 22 patients treated with guselkumab.

Patients receiving risankizumab or guselkumab were classified as the IL-23 inhibitor group, whereas those receiving secukinumab or bimekizumab were classified as the IL-17 inhibitor group. All biologic agents were administered by subcutaneous injection according to their approved standard dosing regimens. Risankizumab was administered at a dose of 150 mg at baseline and week 4, followed by 150 mg every 12 weeks. Guselkumab was administered at a dose of 100 mg at baseline and week 4, followed by 100 mg every 8 weeks. Secukinumab was administered at a dose of 300 mg weekly during the first 5 weeks and every 4 weeks thereafter. Bimekizumab was administered at a dose of 320 mg every 4 weeks through week 16 and every 8 weeks thereafter.

Patients were eligible for inclusion if they were aged ≥18 years, had a diagnosis of moderate-to-severe plaque psoriasis, received continuous treatment with an IL-23 or IL-17 inhibitor, and had complete clinical and laboratory data available at both baseline and the 12-month follow-up.

Patients with incomplete follow-up data, missing laboratory measurements at either time point, or discontinuation or switching of biologic therapy during follow-up were excluded. To minimize potential confounding of lipid-related outcomes, patients with conditions or treatments that could substantially affect lipid metabolism were also excluded. These included obesity, diabetes mellitus, metabolic syndrome, hypertension, clinically significant hepatic or renal disease, nephrotic syndrome, uncontrolled endocrine disorders, active malignancy, pregnancy, smoking, alcohol use, and the use of lipid-lowering or other medications known to substantially affect lipid metabolism. Patients who initiated or modified lipid-lowering therapy during the 12-month follow-up period were also excluded. Dyslipidemia itself, in the absence of lipid-lowering treatment, was not considered an exclusion criterion.

The study was approved by the Clinical Research Ethics Committee of University of Health Sciences Türkiye, Gazi Yaşargil Training and Research Hospital (Approval No. 89; Date: 2 March 2026) and was conducted in accordance with the principles of the Declaration of Helsinki. The requirement for informed consent was waived because of the retrospective nature of the study.

### 4.2. Clinical and Laboratory Assessment

Demographic characteristics, including age and sex, were obtained from electronic medical records. Disease severity was assessed using the Psoriasis Area and Severity Index (PASI) at baseline and after 12 months of treatment.

Fasting serum total cholesterol (TC), low-density lipoprotein cholesterol (LDL-C), high-density lipoprotein cholesterol (HDL-C), triglyceride (TG), and very-low-density lipoprotein cholesterol (VLDL-C) levels were recorded.

Non-HDL cholesterol was calculated as follows:

Non-HDL-C = TC − HDL-C

Remnant cholesterol was calculated as follows:

Remnant cholesterol = TC − LDL-C − HDL-C

The following lipid-derived atherogenic indices were calculated:

- Atherogenic Index of Plasma (AIP) = log10(TG/HDL-C)
- TG/HDL ratio
- Castelli Risk Index-I (CRI-I) = TC/HDL-C
- Castelli Risk Index-II (CRI-II) = LDL-C/HDL-C
- Atherogenic Coefficient (AC) = Non-HDL-C/HDL-C

Treatment-induced changes (Δ) were calculated as the 12-month value minus the baseline value.

### 4.3. Statistical Analysis

Statistical analyses were performed using Jamovi version 2.3.

Continuous variables were expressed as mean ± standard deviation (SD), whereas categorical variables were presented as number (n) and percentage (%). Data normality was assessed using the Shapiro–Wilk test.

Baseline comparisons between the IL-23 and IL-17 inhibitor groups were performed using the independent-samples Student’s *t*-test or the Mann–Whitney U test for continuous variables, as appropriate. Categorical variables were compared using Pearson’s chi-square test.

Within-group comparisons between baseline and month 12 were performed using the paired Student’s *t*-test or the Wilcoxon signed-rank test, according to the distribution of the data.

Treatment-induced changes (Δ values) were compared between the two treatment groups using the independent-samples Student’s *t*-test or the Mann–Whitney U test, as appropriate.

Because baseline total cholesterol and LDL-C levels differed significantly between the treatment groups, additional analysis of covariance (ANCOVA) models were performed for these parameters. Month-12 total cholesterol and LDL-C levels were entered as dependent variables, treatment group as the fixed factor, and the corresponding baseline value as a covariate.

A two-sided *p*-value of <0.05 was considered statistically significant.

## 5. Conclusions

In conclusion, IL-23 inhibitor treatment was associated with greater improvements in several conventional lipid parameters and lipid-derived atherogenic indices compared with IL-17 inhibitor treatment over 12 months, despite comparable improvements in psoriasis severity. These findings suggest that IL-23 and IL-17 inhibition may be associated with differential changes in lipid metabolism. However, these results are based on surrogate lipid-derived markers and should not be interpreted as evidence of differential cardiovascular protection. Larger prospective studies incorporating clinical cardiovascular outcomes are needed to confirm these findings and determine their long-term clinical significance.

## Figures and Tables

**Table 1:** Baseline demographic, clinical, and lipid characteristics of the study groups.

Variable	IL-23 Inhibitors (*n* = 51)	IL-17 Inhibitors (*n* = 57)	*p*-Value
Age (years)	44.1 ± 10.9	45.9 ± 9.81	0.367
Male sex, *n* (%)	33 (64.7)	36 (63.2)	0.867
PASI	19.2 ± 4.57	18.8 ± 4.67	0.701
Total cholesterol (mg/dL)	189 ± 26.2	200 ± 27.6	0.039
LDL cholesterol (mg/dL)	111 ± 25.9	123 ± 23.3	0.013
HDL cholesterol (mg/dL)	44.0 ± 11.5	44.2 ± 7.72	0.929
Non-HDL cholesterol (mg/dL)	145 ± 28.7	156 ± 29.1	0.059
Triglycerides (mg/dL)	154 ± 60.7	164 ± 56.0	0.138
VLDL cholesterol (mg/dL)	30.7 ± 12.1	32.7 ± 11.2	0.111
Atherogenic Index of Plasma (AIP)	0.170 ± 0.241	0.185 ± 0.225	0.721
TG/HDL ratio	3.95 ± 2.43	3.98 ± 2.18	0.714
Castelli Risk Index-I	4.64 ± 1.58	4.68 ± 1.12	0.293
Castelli Risk Index-II	2.71 ± 0.93	2.89 ± 0.77	0.275
Atherogenic Coefficient	3.64 ± 1.58	3.68 ± 1.12	0.293
Remnant cholesterol (mg/dL)	33.8 ± 18.5	32.5 ± 11.2	0.588

Note: Values are presented as mean ± standard deviation (SD) or *n* (%), as appropriate. Continuous variables were compared using the independent-samples Student’s *t*-test for normally distributed data and the Mann–Whitney *U* test for non-normally distributed data. Categorical variables (sex) were compared using Pearson’s chi-square test. A two-sided *p*-value < 0.05 was considered statistically significant.

**Table 2:** Changes in lipid parameters, lipid-related indices, and PASI scores after 12 months of IL-23 inhibitor treatment.

Variable	Baseline (Mean ± SD)	Month 12 (Mean ± SD)	*p*-Value
Total cholesterol (mg/dL)	189.06 ± 26.18	182.12 ± 26.89	0.013
LDL cholesterol (mg/dL)	111.28 ± 25.87	110.81 ± 21.48	0.381
HDL cholesterol (mg/dL)	44.00 ± 11.46	46.39 ± 13.98	0.168
Non-HDL cholesterol (mg/dL)	145.07 ± 28.72	134.62 ± 31.02	<0.001
Triglycerides (mg/dL)	154.06 ± 60.75	136.24 ± 52.04	<0.001
VLDL cholesterol (mg/dL)	30.70 ± 12.07	27.36 ± 10.45	0.005
AIP	0.170 ± 0.241	0.090 ± 0.243	<0.001
TG/HDL ratio	3.95 ± 2.43	3.24 ± 1.67	<0.001
Castelli Risk Index-I	4.64 ± 1.58	4.19 ± 1.08	0.007
Castelli Risk Index-II	2.71 ± 0.93	2.56 ± 0.75	0.043
Atherogenic Coefficient	3.64 ± 1.58	3.19 ± 1.08	0.007
Remnant cholesterol (mg/dL)	33.79 ± 18.51	24.91 ± 15.70	0.001
PASI score	19.16 ± 4.57	0.91 ± 1.09	<0.001

Note: *p*-values were calculated using the Wilcoxon signed-rank test.

**Table 3:** Changes in lipid parameters, lipid-related indices, and PASI scores after 12 months of IL-17 inhibitor treatment.

Variable	Baseline (Mean ± SD)	Month 12 (Mean ± SD)	*p*-Value
Total cholesterol (mg/dL)	199.90 ± 27.62	198.67 ± 34.15	0.123
LDL cholesterol (mg/dL)	123.23 ± 23.33	121.94 ± 28.85	0.144
HDL cholesterol (mg/dL)	44.16 ± 7.72	45.34 ± 7.82	0.188
Non-HDL cholesterol (mg/dL)	155.73 ± 29.13	153.33 ± 35.55	0.080
Triglycerides (mg/dL)	163.54 ± 55.99	164.09 ± 61.35	0.100
VLDL cholesterol (mg/dL)	32.74 ± 11.19	32.95 ± 12.36	0.067
AIP	0.185 ± 0.225	0.179 ± 0.193	0.066
TG/HDL ratio	3.98 ± 2.18	3.80 ± 1.76	0.034
Castelli Risk Index-I	4.68 ± 1.12	4.51 ± 1.11	0.138
Castelli Risk Index-II	2.89 ± 0.77	2.78 ± 0.86	0.054
Atherogenic Coefficient	3.68 ± 1.12	3.51 ± 1.11	0.139
Remnant cholesterol (mg/dL)	32.50 ± 11.24	31.39 ± 12.09	0.481
PASI score	18.82 ± 4.67	0.86 ± 0.97	<0.001

**Table 4:** Comparison of changes (Δ) between IL-23 and IL-17 treatment groups.

Variable	IL-23 (Δ, Mean ± SD)	IL-17 (Δ, Mean ± SD)	*p*
ΔTC	−6.94 ± 20.3	−1.23 ± 18.5	0.001
ΔLDL	−0.46 ± 20.1	−1.29 ± 17.8	0.117
ΔHDL	2.40 ± 14.5	1.18 ± 6.80	0.233
ΔNon-HDL	−10.4 ± 24.7	−2.41 ± 19.4	<0.001
ΔTG	−17.8 ± 44.6	0.54 ± 58.2	<0.001
ΔVLDL	−3.35 ± 9.10	0.21 ± 11.6	<0.001
ΔAIP	−0.079 ± 0.188	−0.005 ± 0.169	<0.001
ΔTG/HDL	−0.703 ± 1.91	−0.180 ± 2.18	<0.001
ΔCRI-I	−0.449 ± 1.50	−0.171 ± 0.852	0.002
ΔCRI-II	−0.154 ± 0.786	−0.108 ± 0.596	0.015
ΔAC	−0.449 ± 1.50	−0.171 ± 0.852	0.002
ΔRemnant	−8.87 ± 21.5	−1.12 ± 12.6	<0.001
ΔPASI	−18.2 ± 4.60	−18.0 ± 4.54	0.739

Note: Data are presented as mean ± SD. The Mann–Whitney U test was used for all variables except ΔPASI, which was analyzed using Student’s *t*-test.

## Data Availability

The raw data supporting the conclusions of this article will be made available by the authors on request.
